# HTR2B and SLC5A3 Are Specific Markers in Age-Related Osteoarthritis and Involved in Apoptosis and Inflammation of Osteoarthritis Synovial Cells

**DOI:** 10.3389/fmolb.2021.691602

**Published:** 2021-06-16

**Authors:** Xin Lu, Yu Fan, Mingxia Li, Xiao Chang, Jun Qian

**Affiliations:** ^1^ Department of Orthopaedics, Peking Union Medical College Hospital, Chinese Academy of Medical Sciences and Peking Union Medical College, Beijing, China; ^2^ The Institute of Basic Medical Sciences, Chinese Academy of Medical Sciences and Peking Union Medical College, Beijing, China

**Keywords:** osteoarthritis, synovial tissues, weighted correlation network analysis, HTR2B, SLC5A3, apoptosis, inflammation

## Abstract

**Objective:** Osteoarthritis (OA) is a heterogeneous age-related disease, which is badly difficult to cure due to its complex regulatory networks of pathogenesis. This study explored OA-specific genes in synovial tissues and validated their roles on apoptosis and inflammation of OA synovial cells.

**Methods:** Weighted correlation network analysis (WGCNA) was employed to explore OA-related co-expression modules in the GSE55235 and GSE55457 datasets. Then, this study screened OA-specific genes. After validation of these genes in the GSE12021 and GSE32317 datasets, HTR2B and SLC5A3 were obtained. Their expression was detected in human OA and healthy synovial tissues by RT-qPCR and western blot. OA rat models were constructed by anterior cruciate ligament transection (ACLT) operation. In OA synovial cells, HTR2B and SLC5A3 proteins were examined via western blot. After transfection with sh-HTR2B or sh-SLC5A3, apoptosis and inflammation of OA synovial cells were investigated by flow cytometry and western blot.

**Results:** A total of 17 OA-specific DEGs were identified, which were significantly enriched in inflammation pathways. Among them, HTR2B and SLC5A3 were highly expressed in end-than early-stage OA. Their up-regulation was validated in human OA synovial tissues and ACLT-induced OA synovial cells. Knockdown of HTR2B and SLC5A3 restrained apoptosis and increased TGF-β and IL-4 expression as well as reduced TNF-α and IL-1β expression in OA synovial cells.

**Conclusion:** Collectively, this study identified two OA-specific markers HTR2B and SLC5A3 and their knockdown ameliorated apoptosis and inflammation of OA synovial cells.

## Introduction

Osteoarthritis (OA) is an age-related chronic joint disease, characterized by degenerated cartilage erosion, osteophyte formation, subchondral bone modification as well as synovitis (Hunter et al., 2020; Kolasinski et al., 2020; Xie et al., 2020). OA is a heterogeneous and multifactorial disease, involving genetic, biological and immunologic factors (Ratneswaran et al., 2020). Its main manifestations are joint pain, swelling, stiffness, deformity, and dysfunction, which seriously endanger the health of middle-aged and elderly people (Woolf and Pfleger, 2003; Latourte et al., 2020; Rim et al., 2020). It has been estimated that about 10% of men and 18% of women over 65 have varying degrees of OA (Lie et al., 2019). With the aging of the global population, the incidence of OA is on the rise, but no targeted drugs have been found to effectively cure this disease (Mandl, 2019).

Except the cartilage damage as the most remarkable alterations, synovial tissues, joint ligaments and subchondral bone are affected (Abramoff and Caldera, 2020). Previous views believed that changes in the biomechanical and chemical factors of articular cartilage might lead to OA, but the latest research has found that pathological changes in the synovium may be an important factor affecting OA (Zhang et al., 2019). Weighted gene co-expression network analysis (WGCNA) is an algorithm based on high-throughput gene expression profiling (Langfelder and Horvath, 2008). It is widely used in the identification of gene co-expression networks of various diseases to reveal the correlation between genes and to find significantly related gene modules (Niu et al., 2019; Song et al., 2019; Li et al., 2020). With the help of WGCNA algorithm, gene modules can be constructed, and the pivot genes in related gene modules can be studied. Here, this study retrieved the gene expression profiles of OA synovial tissues from the Gene Expression Omnibus (GEO) database. WGCNA was employed to explore OA-specific genes. Among them, HTR2B and SLC5A3 were confirmed to be up-regulated in human OA synovial tissues and anterior cruciate ligament transection (ACLT)-induced OA synovial cells. After silencing their expression, apoptosis and inflammation of OA synovial cells were distinctly ameliorated. Thus, this study may provide novel therapeutic targets against OA progression.

## Materials and Methods

### Acquisition of Datasets

Microarray expression profiles of OA were retrieved from the GEO (https://www.ncbi.nlm.nih.gov/gds/) database, including GSE55235, GSE55457, GSE12021 and GSE32317. Synovial tissues from 10 osteoarthritic joint and 10 healthy joint were retrieved from the GSE55235 dataset (Woetzel et al., 2014). The GSE55457 dataset contained 10 normal and 10 OA synovial membrane samples (Woetzel et al., 2014). The GSE12021 dataset included 20 osteoarthritic and 11 normal synovial membrane specimens (Huber et al., 2008). The GSE32317 dataset was comprised of 10 early and 9 end-stage OA synovial membrane samples. The GSE55235 and GSE55457 datasets were employed for differential expression analysis and WGCNA. The GSE12021 and GSE32317 datasets were used for validating the OA-specific DEGs.

### Differential Expression Analysis

The gene expression matrix was analyzed through limma package v3.9.19 (Ritchie et al., 2015). Differentially expressed genes (DEGs) were screened between OA and normal synovial tissues. Genes with |fold change (FC)| > 1.5 and false discovery rate (FDR) < 0.05 were considered differentially expressed in OA. The results of DEGs were visualized into volcano and heatmap plots.

### WGCNA

WGCNA package was employed to find co-expression modules of highly corelated genes across specimens from the GSE55235 and GSE55457 datasets (Langfelder and Horvath, 2008). The correlation coefficients between two genes were calculated, and then a similarity matrix was constructed. The formula was as follows: $S_{mn}=cor\left(X_{m},X_{n}\right)$, $S=\left[S_{mn}\right]$, where $S_{mn}$ represents the Pearson correlation coefficient between gene m and gene n, and S indicates the similarity matrix. To make the network conform to the non-scale network, the appropriate weight value was selected using the pickSoftThreshold function. To eliminate the errors caused by background noise and pseudo-correlation, the adjacency matrix was converted to the topological overlap matrix (TOM). Furthermore, the function dissTom = 1–TOM was used to inverse the topological overlap matrix to obtain the dissimilarity matrix. Using the function hclust, we presented hierarchical clustering of dissimilar matrices. The dynamic tree cut method was employed to cut the gene clustering tree. The minModuleSize was set as 30. This process can merge genes with similar expression patterns in the same branch, and each branch represents a co-expression module. Genes with similar expression patterns are considered to have similar biological functions. A module is a collection of genes with highly similar expression patterns. To in-depth study the modules that were highly related to OA, the correlation coefficients between module eigengene (ME) and OA were calculated. The larger the correlation coefficient, the higher the correlation between the module and OA. The threshold value of the correlation coefficient defined in this study was 0.65, that is, any module with a correlation coefficient higher than 0.65 would be defined as an OA-specific module. Gene significance (GS) is the Pearson correlation coefficient between the expression of each gene and OA. Module membership (MM) is the correlation coefficient between each gene in the module and the ME. GS and MM were calculated to measure the importance of genes in the module.

### Protein-Protein Interaction

After intersection of DEGs and the genes in the OA-related modules, OA-related DEGs were identified and uploaded onto the STRING database v11 (https://string-db.org/) (Szklarczyk et al., 2017). Parameters were set to default. Then, a PPI network was established. Furthermore, the correlation between OA-related DEGs was evaluated by Spearson analysis.

### Functional Enrichment Analysis

Biological functions of OA-related DEGs were analyzed via clusterProfiler package, including Gene Ontology (GO) and Kyoto Encyclopedia of Genes and Genomes (KEGG) enrichment analyses (Yu et al., 2012). GO contained biological process (BP), cellular component (CC) and molecular function (MF). Terms with adjusted *p*-value < 0.05 indicated significant enrichment.

### Specimens

A total of 12 healthy synovial tissues from individuals during the operation of traffic accidents and 15 OA synovial tissues were collected in the Peking Union Medical College Hospital between January 2019 to January 2020. The synovial tissues were examined macroscopically and microscopically. OA synovial samples were obtained from patients that underwent total knee arthroplasty. Each OA patient was diagnosed in line with the criteria of the American College of Rheumatology Diagnostic Subcommittee for OA. Each participant provided written informed consent and this research gained the approval of the ethics committee of Peking Union Medical College Hospital (2019016).

### Real-Time Quantitative Polymerase Chain Reaction

Total RNA was extracted from synovial tissues by the Trizol reagent (Beyotime, Shanghai, China). The absorbance of RNA was measured and the A260/A280 ratio was used to evaluate the purity of RNA. RT-qPCR was presented based on the SYBR qPCR mix manual. GAPDH was used as an internal reference. The relative expression of the target genes was analyzed using the 2^−ΔΔCt^ method. The primer sequences were as follows: HTR2B: 5′-TGA​TTT​GCT​GGT​TGG​ATT​GTT​TG-3’ (forward), 5′-ATG​GAT​GCG​GTT​GAA​AAG​AGA​A-3’ (reverse); SLC5A3: 5′-AGC​ACC​GTG​AGT​GGA​TAC​TTC-3’ (forward), 5′-CCC​TGA​CCG​GAT​GTA​AAT​TGG-3’ (reverse); GAPDH: 5′-CTG​GGC​TAC​ACT​GAG​CAC​C-3’ (forward), 5′-AAG​TGG​TCG​TTG​AGG​GCA​ATG-3’ (reverse).

### Western Blot

Total protein was extracted from tissues by RIPA lysis buffer (Beyotime), 10 μL PMSF and 10 μL phosphatase inhibitor. SDS-PAGE gel electrophoresis was utilized to separate the extracted protein. The protein was transferred to polyvinylidene fluoride membrane. After blocking with 50 g/L BAS for 1 h, the membrane was incubated with primary antibodies against HTR2B (1/3000; ab227722; Abcam, Cambridge, MA, United States), SLC5A3 (1/1000; ab113245), GAPDH (1/3000; ab8245), TNF-α (1/1000; ab215188), IL-1β (1/1000; ab200478), TGF-β (9ab208156) and IL-4 (1/1000; ab34277) at 4°C overnight. After washing the membrane with TBST, the membrane was incubated with secondary antibody (1/5000; ab7097) for 1.5 h. The ultra-sensitive ECL chemiluminescence kit was used to configure the luminescence buffer, and the band was developed.

### Animal Models

Healthy 3-month-old SD female rats (weighing 200–250 g) that were purchased from Beijing Vital River Laboratory Animal Technology Co., Ltd. (China), were bred adaptively for 1 week. All the tested rats were kept in separate cages in an animal room under natural light at a room temperature of 20–24°C. A rat model of knee arthritis was established by cutting the right ACLT. The rats were fasted 12 h before modeling, and 2% sodium pentobarbital was used for intraperitoneal anesthesia at a dosage of 30 mg/kg. After the anesthesia, the rats were shaved before surgery, routinely disinfected, and draped in front of the knee. A median incision was made, and the skin subcutaneous tissue was found to find the white patellar tendon, and cut the knot bundle along its medial side. At the same time, we made the knee joint hyperextension and flexed the knee joint to find the anterior cruciate ligament, cut off with ophthalmology, and restored its normal straight position. After that, the patella was repositioned, the joint capsule, subcutaneous tissue and skin were sutured layer by layer, disinfected again before suture, and a drawer experiment was performed after the operation to verify that the ACL was indeed cut off. In the sham group, only the right knee joint cavity was cut, the anterior cruciate ligament was not cut, and the knee joint was sutured directly. The left knee joint was not operated on. After operation, each rat was intramuscularly injected with penicillin (400,000 units) once a day for 3 consecutive days to prevent infection. Then, rats continued to be fed for 1 week after modeling. This animal experiment followed the Guide of the Management and Use of Laboratory Animals issued by the National Institutes of Health and gained the approval of the Institutional Animal Care and Use Committee (IACUC) of Peking Union Medical College Hospital (2019016).

### Culture of Synovial Cells

The synovial tissue removed during the operation was cut into pieces under aseptic conditions, soaked in D-Hank’s solution and rinsed 3 times. After separating and removing mixed adipose tissues and a small amount of muscle tissues, samples were cut into 1 mm^3^ tissue pieces. Then, samples were centrifuged at 2500 r/min for 6 min and washed twice. The supernatant was aspirated and discarded. 100 μL fetal bovine serum (FBS) was added and mixed, suck out with a pipette and place it in a culture flask, with each small piece about 5 mm apart. The culture bottle was gently turned over, with the bottom of the bottle facing upwards. 3 ml culture medium containing 20% FBS was injected into the bottle, and placed it in a CO_2_ incubator (37°C, 5% CO_2_) to grow adherently. After 5 h, the culture bottle was turned upright. The medium for the first time was changed after standing for 4–5 days, and the cell growth and adhesion was observed under an inverted microscope. Subculture was carried out when the primary cultured synovial cells were 85–95% of the bottom of the culture flask. This experiment was generally 9–11 days in line with the passage. Subculture inoculation at a ratio of 1:2 or 1:3, this was the first generation of synovial cells. When the culture continued to cover 85–95% of the bottom of the bottle, synovial cells were passaged again at a ratio of 1:3.

### Transfection

The shRNAs against HTR2B and SLC5A3 were completed by Shanghai GenePharma Company (China). Double-stranded DNA fragments were cut to sticky ends and hairpin sequences with BHSI and BamHI restriction enzymes *in vitro*. The fragment was ligated to the pGPHI/GFP/Neo vector, transformed into *E. coli* DH5α for propagation, and then amplified and screened. The second-generation synovial cells were seeded in a 6-well plate (2 × 10^5^ cells/well). Then, the medium containing the lentivirus solution was added to each well. The lentivirus carrying sh-HTR2B and sh-SLC5A3 with a virus titer of 3 × 10^8^ was transfected into synovial cells. After 48 h of transfection, the inhibition effect was detected by RT-qPCR.

### Flow Cytometry

The Annexin V-FITC/PI apoptosis kit (APOAF-20TST; Sigma-Aldrich, United States) was used for detecting cell apoptosis. Cells were added with 5 μL Annexin V-FITC and 5 μL PI successively after suspending. After mixing, cells were incubated at room temperature and avoided light for 15 min. Apoptosis was detected by flow cytometry.

### Statistical Analyses

Statistical analyses were presented using R language v3.6.1 and GraphPad Prism v8.0 (GraphPad, San Diego, CA). Data are presented as mean ± standard deviation. Comparisons between groups were assessed through unpaired student’s t test or one-way analysis of variance. *p*-value < 0.05 was indicative of statistical significance.

## Results

### Screening DEGs in Osteoarthritic Synovial Tissues

**FIGURE 1:**
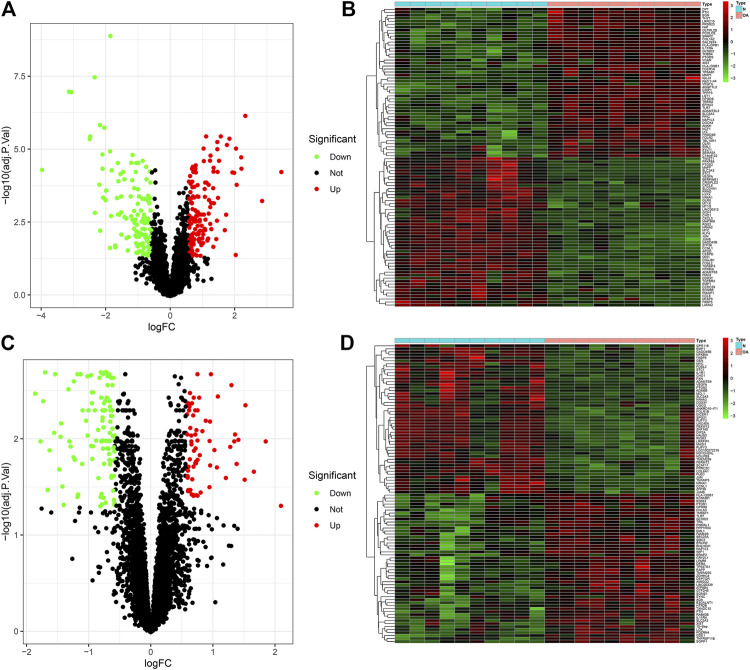
Identifying DEGs in osteoarthritic and healthy synovial tissues in the GSE55235 and GSE55457 datasets. **(A,B)** Volcano and heatmap for visualizing up- (red) and down-regulated (green) genes in osteoarthritic compared to healthy synovial tissues in the GSE55235 dataset. Black spots indicate non-DEGs. **(C,D)** Volcano and heatmap showing up- (red) and down-regulated (green) genes between OA and control synovial tissues in the GSE55457 dataset.

This study employed the GSE55235 and GSE55457 datasets for differential expression analysis in OA. Under the criteria of |FC| > 1.5 and FDR < 0.05, 145 genes were up-regulated and 133 genes were down-regulated in 10 OA synovial tissues compared to 10 healthy synovial tissues in the GSE55235 dataset (Figure 1A). The specific information of DEGs was listed in Supplementary Table S1. Heat map visualized the expression of each DEG in each OA and healthy synovial tissue (Figure 1B). A distinct difference in DEGs was found between OA and healthy samples. In the GSE55457 dataset, we screened 69 up- and 104 down-regulated genes in 10 OA than 10 normal synovial samples (Figure 1C; Supplementary Table S2). These DEGs could differentiate OA from normal specimens (Figure 1D).

### Establishment of OA-Related Co-Expression Modules in the GSE55235 Dataset

**FIGURE 2:**
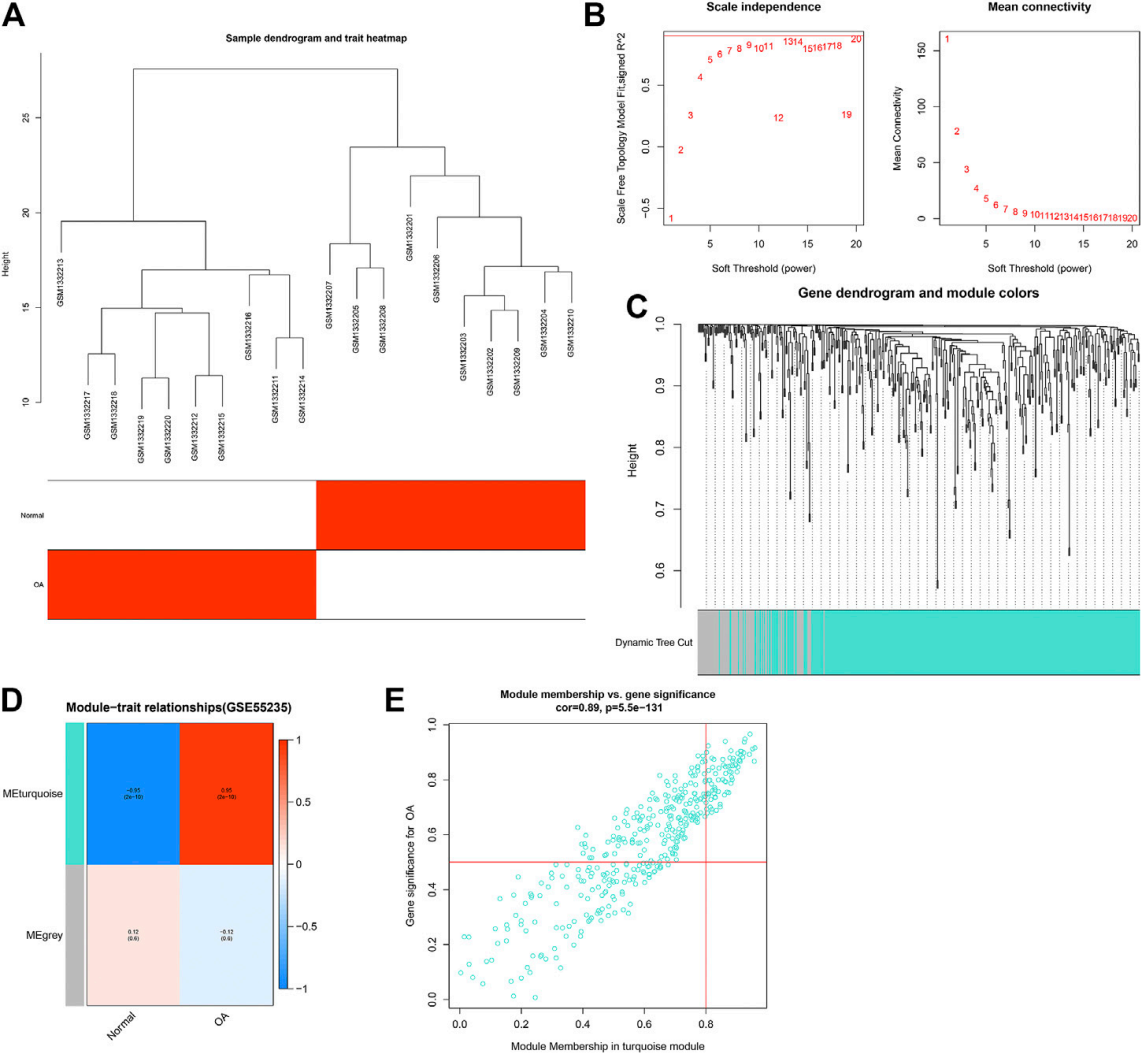
Establishment of co-expression modules for OA in the GSE55235 dataset. **(A)** Heatmap for sample dendrogram and clinical traits. **(B)** Determination of soft-thresholding power (β). Under different β values, scale independence and mean connectivity were determined. **(C)** Gene dynamic cutting cluster tree. Each color represents a module. **(D)** Heatmap for correlations of modules with clinical traits. Each row represents a module, and each column represents a trait. The numbers in the rectangular box represent the correlation coefficient between the module and the trait and the corresponding *p* value. Red represents positive correlation, and blue represents negative correlation. The darker the color, the stronger the correlation. **(E)** Scatter plots for the correlation between GS and MM in the turquoize module.

WGCNA was applied for finding OA-related co-expression modules. We firstly detected outlier samples in the GSE55235 dataset. As shown in Figure 2A, there was no outlier. The gene clustering tree of each sample can correspond well to the sample. According to Figure 2B, the soft threshold β = 20 was chosen to construct the scale-free co-expression network. The dynamic cutting tree method was used to merge the modules with similar expression patterns. As a result, 2 co-expression modules were obtained (Figure 2C). Different colors represented different modules. The number of genes in the module was allocated according to their expression level to perform a correlation clustering, and the genes with a higher clustering degree were allocated to a module. There were 380 genes in the turquoize module (Supplementary Table S3). The gray module contained a set of genes that were not assigned to the turquoize module. The turquoize module was highly correlated to OA (*r* = 0.95 and *p* = 2e-10; Figure 2D). As shown in Figure 2E, genes in the turquoize module exhibited a strong correlation to OA (*r* = 0.89, *p* = 5.5e-131).

### Construction of OA-Related Co-Expression Modules in the GSE55457 Dataset

**FIGURE 3:**
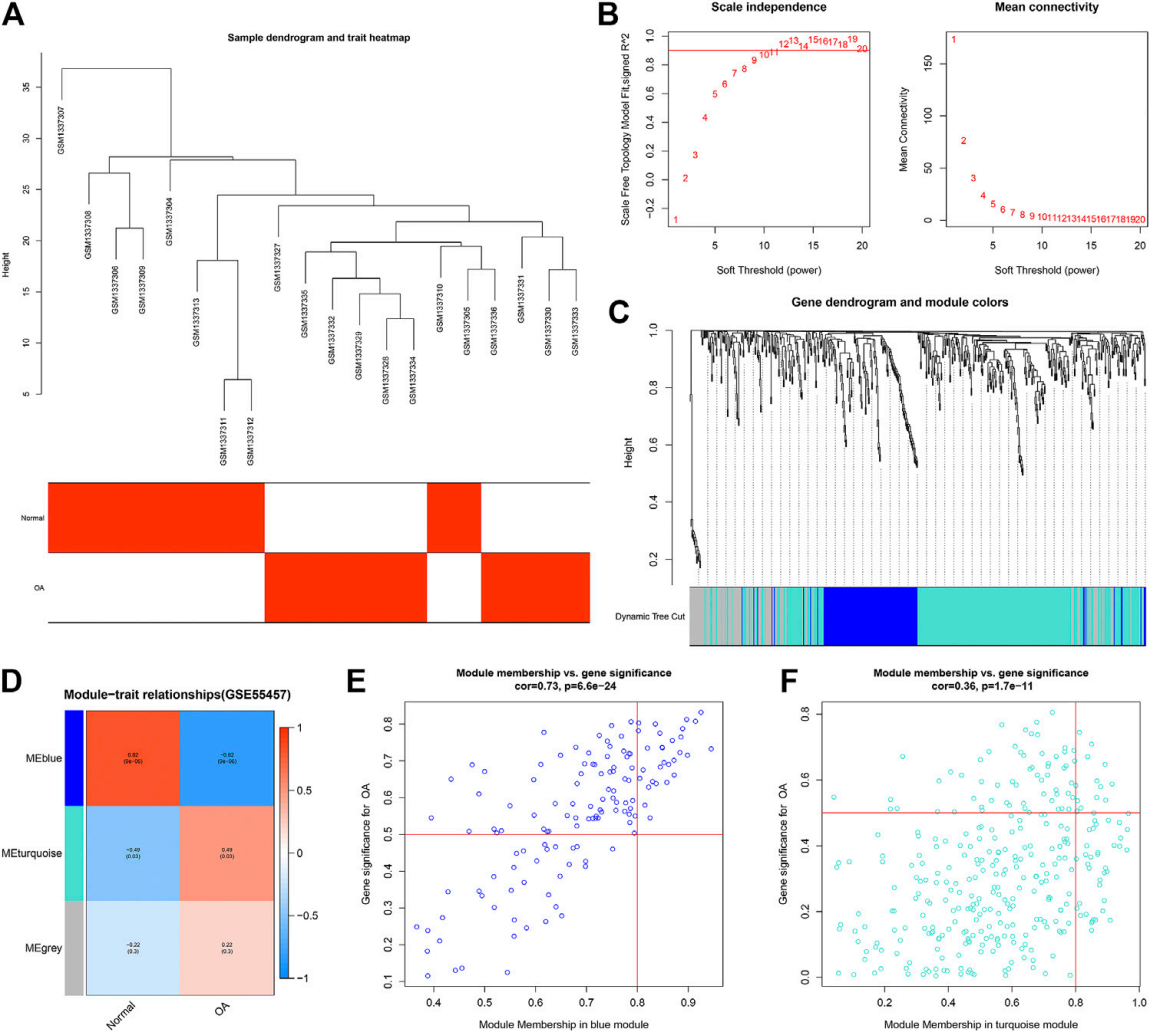
Construction of co-expression modules for OA in the GSE55457 dataset. **(A)** Heatmap showing sample dendrogram and clinical traits. **(B)** Determination of the appropriate soft-thresholding power (β). **(C)** Gene dendrogram. Each color expresses a module. **(D)** Heatmap for correlations between modules and clinical traits. **(E,F)** Scatter plots for the correlations between GS and MM in the blue and turquoize modules.

For clustering of 10 OA and 10 normal synovial samples in the GSE55457 dataset, no outlier sample was found (Figure 3A). With the soft threshold β = 20, the network was scale-free (Figure 3B). Totally, 3 co-expression modules were merged (Figure 3C). The genes in the gray module cannot be classified into any other modules. Among them, the blue module was negatively associated with OA (*r* = −0.82 and *p* = 9e-06) while the turquoize module was positively correlated to OA (*r* = 0.49 and *p* = 0.03; Figure 3D). The genes in the blue (*r* = 0.73 and *p* = 6.6e-24; Figure 3E) and turquoize (*r* = 0.36 and *p* = 1.7e-11; Figure 3F) modules displayed significant correlations to OA. Supplementary Table S4 listed all genes in the turquoize module.

### Identifying OA-Specific DEGs and Their Functions

**FIGURE 4:**
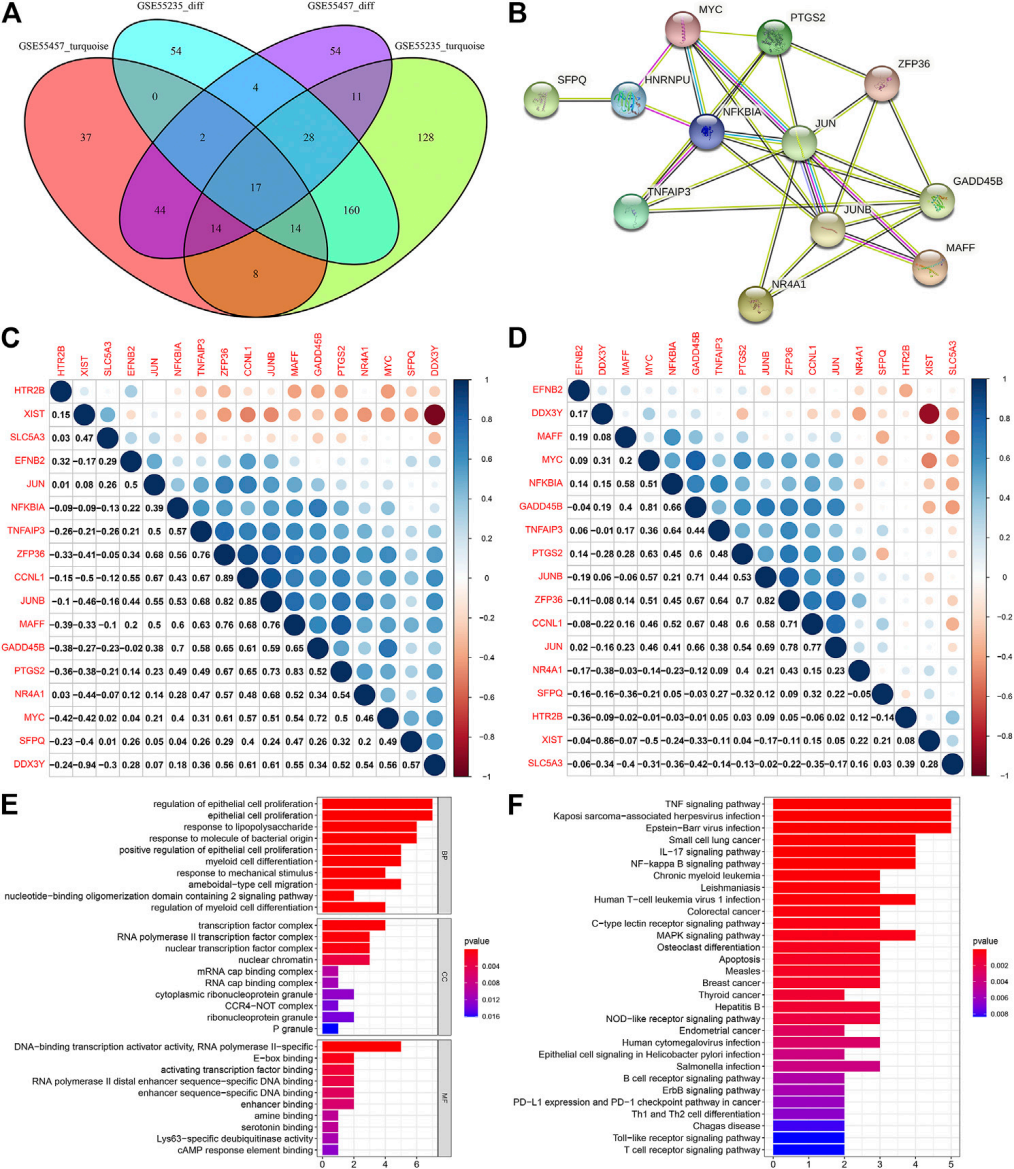
Identifying OA-specific DEGs and their functions. **(A)** Venn diagram for OA-specific DEGs by intersections of genes in the turquoize modules and DEGs. **(B)** A PPI network based on OA-specific DEGs. **(C,D)** Heatmap for the correlations between OA-specific DEGs in the **(C)** normal and **(D)** OA samples. Blue represents positive correlation and red represents negative correlation. The bigger the circle, the stronger the correlation. **(E,F)** GO and KEGG enrichment analysis results of OA-specific DEGs. The ordinate represents the GO entry or the KEGG pathway, and the abscissa represents the percentage of the enriched genes in the module. The length of the rectangle represents the number of enriched genes, and the color of the rectangle represents the *p* value.

We identified 17 OA-specific DEGs by intersection of genes in the turquoize modules and DEGs, including CCNL1, DDX3Y, EFNB2, GADD45B, HTR2B, JUN, JUNB, MAFF, MYC, NFKBIA, NR4A1, PTGS2, SFPQ, SLC5A3, TNFAIP3, XIST and ZFP36 (Figure 4A). Genes with similar expression patterns may have the same biological functions. The modules constructed through co-expression networks could be largely related to specific biological tissues, and specific biological tissues could be highly synergistic with specific life activities. Here, we constructed a PPI network based on these OA-specific DEGs. There were 12 nodes in this network (Figure 4B). Both in the normal and OA samples, their closely interactions between them were found, as shown in Figures 4C,D. To further analyze the biological functions of OA-specific modules, this study conducted enrichment analysis on OA-specific DEGs. We found that they were mainly enriched in proliferation and differentiation of epithelial cells and myeloid cells (Figure 4E). Furthermore, they were primarily associated with inflammation-related pathways such as TNF, IL-17, B cell receptor, T cell receptor and Toll-like receptor signaling pathways as well as Th1 and Th2 cell differentiation (Figure 4F).

### Validation of OA-Specific DEGs in Independent Datasets

**FIGURE 5:**
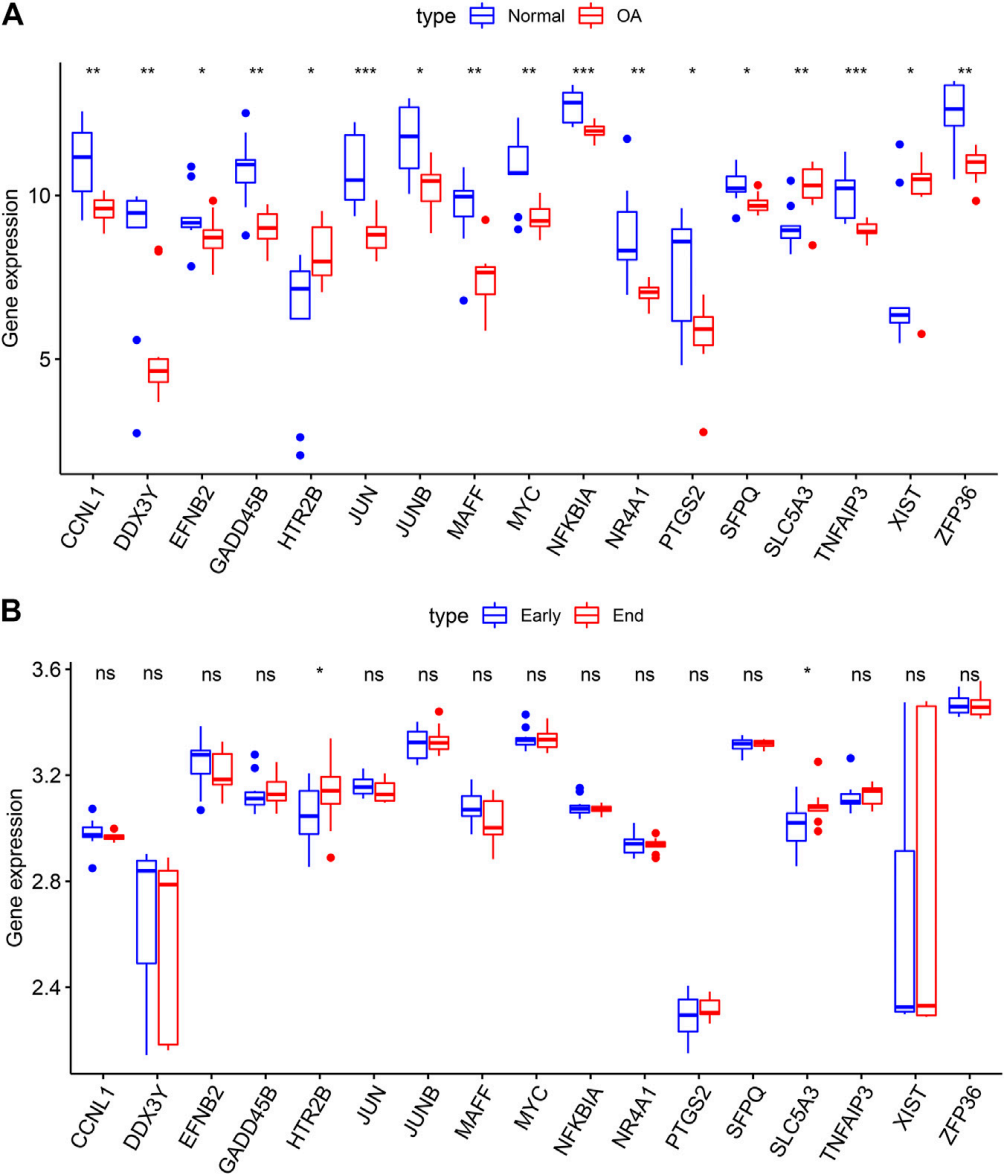
Validation of OA-specific DEGs in the GSE12021 and GSE32317 datasets. **(A)** Box plots for the expression patterns of these 17 OA-specific DEGs in normal and OA synovial tissues in the GSE12021 dataset. **(B)** Box plots for validation of the expression of the OA-specific DEGs in early- and end-stage OA synovial membrane samples in the GSE32317 dataset. Ns: not significant; *p < 0.05; **p < 0.01; ***p < 0.001.

We further validated the expression of the OA-specific DEGs in the GSE12021 dataset. Our results confirmed that CCNL1, DDX3Y, EFNB2, GADD45B, JUN, JUNB, MAFF, MYC, NFKBIA, NR4A1, PTGS2, SFPQ, TNFAIP3 and ZFP36 were lowly expressed in OA compared to normal synovial samples in the GSE12021 dataset (*p* < 0.05; Figure 5A). Meanwhile, HTR2B, SLC5A3 and XIST were highly expressed in OA than normal synovial tissues. Furthermore, we assessed the expression patterns of these OA-specific DEGs in 10 early- and 9 end-stage OA synovial membrane samples from the GSE32317 dataset. As a result, HTR2B and SLC5A3 displayed higher expression levels in end-stage than early-stage OA samples (*p* < 0.05; Figure 5B). These data were indicative that HTR2B and SLC5A3 could participate in OA progression.

### Verification of HTR2B and SLC5A3 Expression in OA Synovial Tissues

**FIGURE 6:**
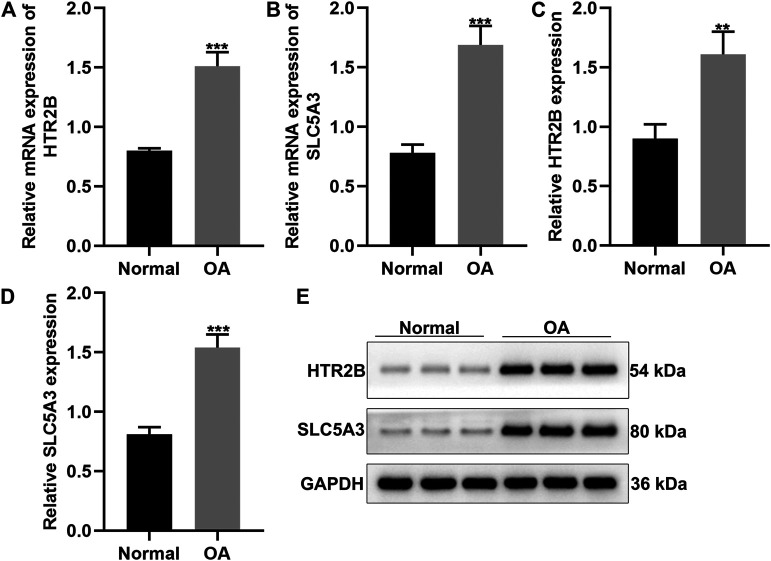
Validation of HTR2B and SLC5A3 expression in OA synovial tissues. **(A,B)** RT-qPCR for the mRNA expression of HTR2B and SLC5A3 in OA and healthy synovial tissues. **(C–E)** Western blot for detecting the expression of HTR2B and SLC5A3 proteins in OA and healthy synovial tissues. **p < 0.01; ***p < 0.001.

Here, we gathered 15 OA and 12 healthy synovial tissues. Our results confirmed that HTR2B and SLC5A3 mRNAs were significantly up-regulated in OA than normal synovial tissues (*p* < 0.05; Figures 6A,B). Also, their overexpression was detected in OA by western blot (Figures 6C–E).

### HTR2B and SLC5A3 Are Overexpressed in Synovial Cells of OA Rats

**FIGURE 7:**
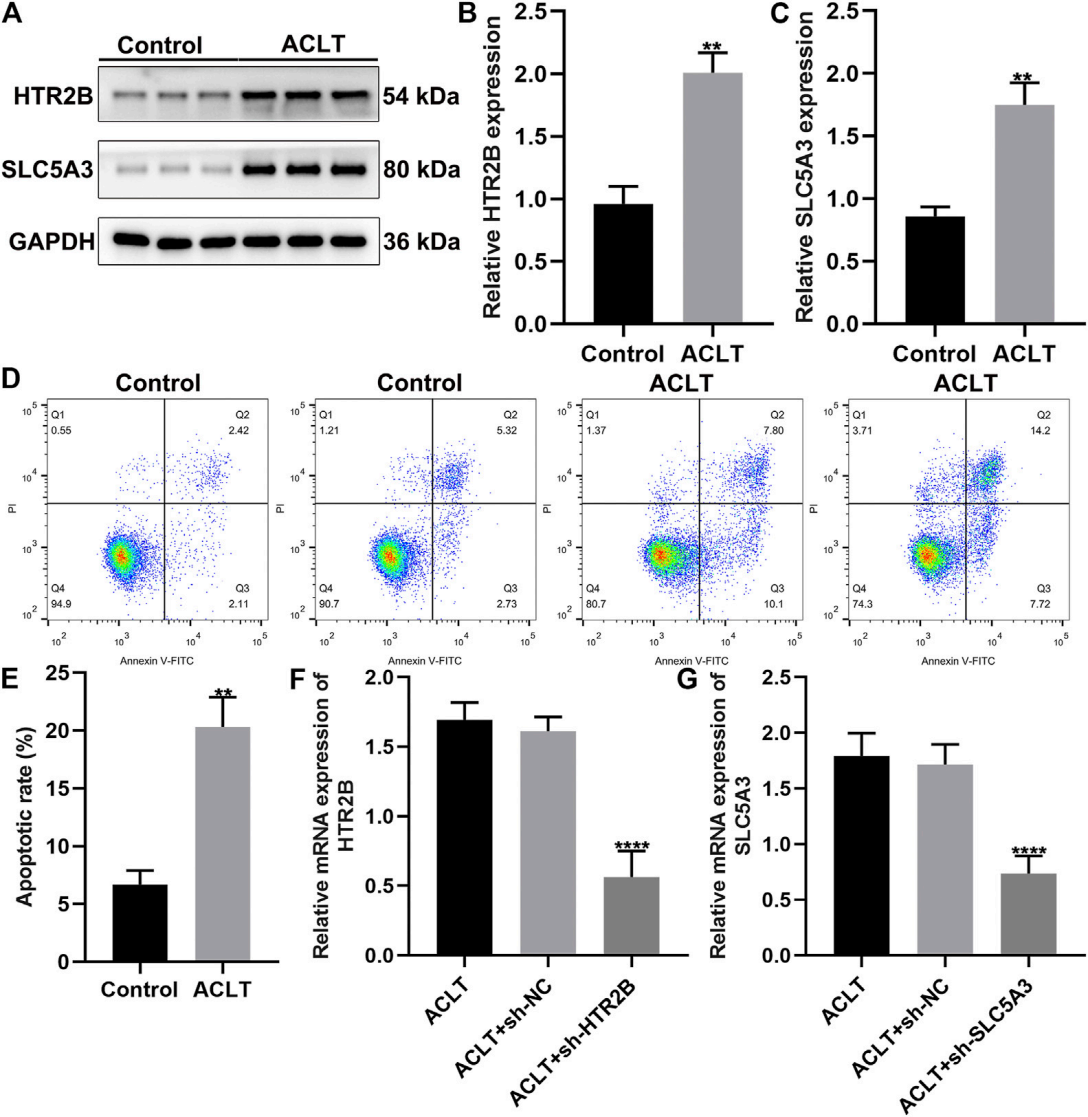
Up-regulation of HTR2B and SLC5A3 in synovial cells of OA rats. **(A–C)** Western bot for detection of the expression of HTR2B and SLC5A3 proteins in synovial cells from sham-operation control and OA rats. **(D,E)** Flow cytometry for the apoptotic levels of control and OA synovial cells. **(F,G)** RT-qPCR for examining the mRNA expression of HTR2B and SLC5A3 in OA synovial cells transfected with sh-HTR2B or sh-SLC5A3. **p < 0.01; ****p < 0.0001.

This study constructed ACLT-induced OA rat models. Then, synovial cells from sham-operation control group and ACLT group were separated and cultured. After 4 days of primary culture, fibroblast-like growth of synovial cells was seen under the inverted microscope when the medium was changed for the first time. The cells divided and proliferated rapidly, and were arranged in a long spindle polar orientation. After the 9th day of culture, the cells were almost all over the bottom of the bottle. After passage, the synovial cells began to adhere to the wall after 2 h, and could fully adhere to the wall after 12 h. The growth rate was faster, and the shape was mainly fusiform. The bottom of the bottle could be grown on the third day. The growth of synovial cells after the second generation was relatively stable, and there remained basically only B-type cells that grown adherently. We examined the expression of HTR2B and SLC5A3 in control and ALCT-induced OA synovial cells. As a result, HTR2B and SLC5A3 proteins were both overexpressed in OA synovial cells compared to control synovial cells (*p* < 0.05; Figures 7A–C). Flow cytometry was utilized for detecting apoptotic levels of synovial cells. The results showed that the apoptotic levels were distinctly elevated in OA synovial cells than controls (*p* < 0.05; Figures 7D,E). To silence HTR2B and SLC5A3 expression, shRNAs against HTR2B and SLC5A3 were separately transfected into OA synovial cells. RT-qPCR results confirmed the successful knockdown of HTR2B and SLC5A3 in OA synovial cells (*p* < 0.05; Figures 7F,G).

### Knockdown of HTR2B or SLC5A3 Restrains Apoptosis of OA Synovial Cells

**FIGURE 8:**
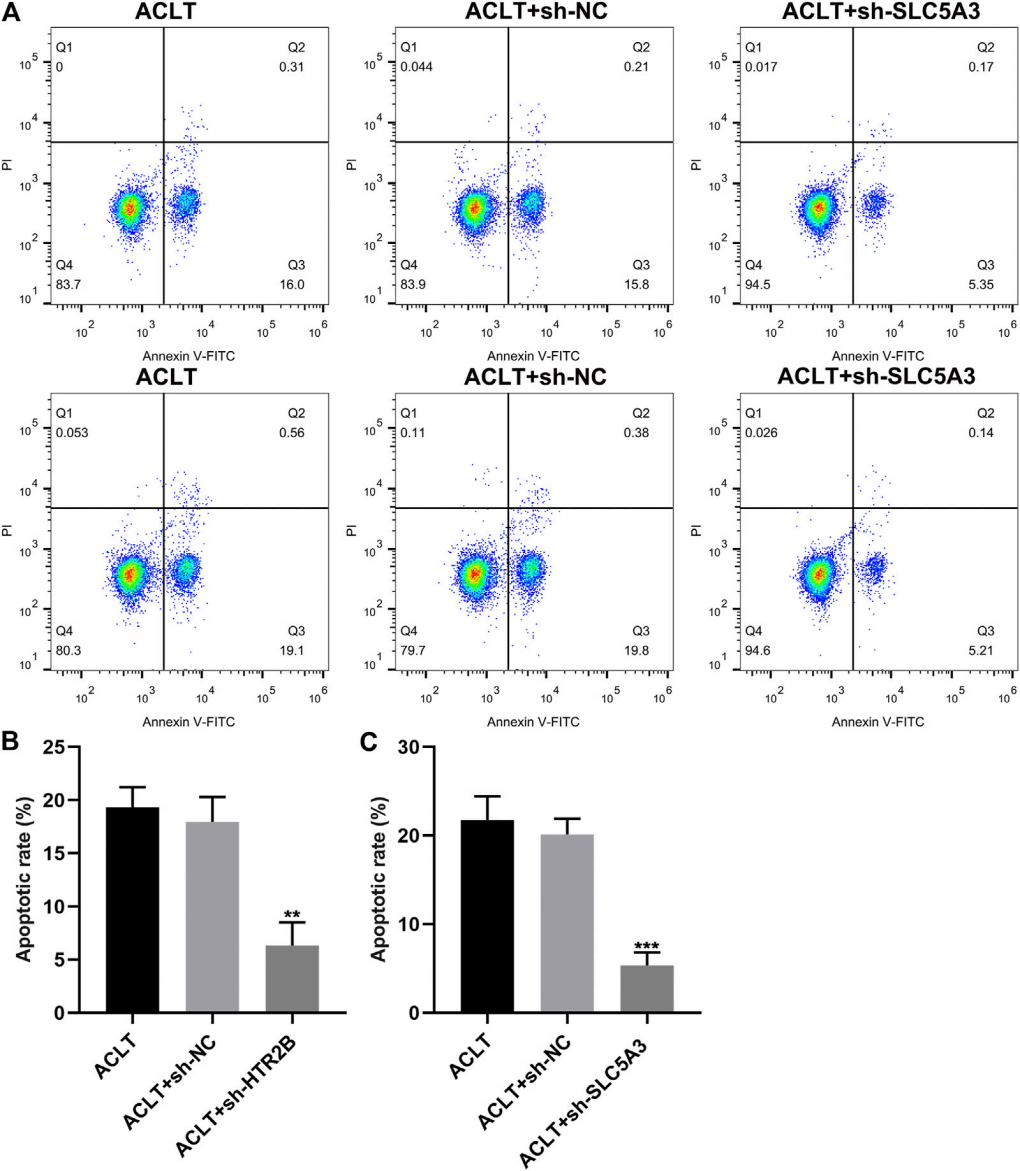
The effects of HTR2B or SLC5A3 knockdown on apoptotic levels of OA synovial cells. **(A)** Representative images of flow cytometry of OA synovial cells transfected with sh-HTR2B or sh-SLC5A3. **(B,C)** Determination of apoptotic levels of OA synovial cells transfected with sh-HTR2B or sh-SLC5A3. **p < 0.01; ***p < 0.001.

Apoptosis of OA synovial cells was assessed by flow cytometry. No distinct difference was investigated between ACLT group and ACLT + sh-NC group (*p* < 0.05; Figures 8A–C). There were lowered apoptotic levels in ACLT + sh-HTR2B or ACLT + sh-SLC5A3 group in comparison to ACLT + sh-NC group. Hence, silencing HTR2B or SLC5A3 could exert inhibitory effects on apoptosis of OA synovial cells.

### Knockdown of HTR2B or SLC5A3 Suppresses Inflammatory Response of OA Synovial Cells

**FIGURE 9:**
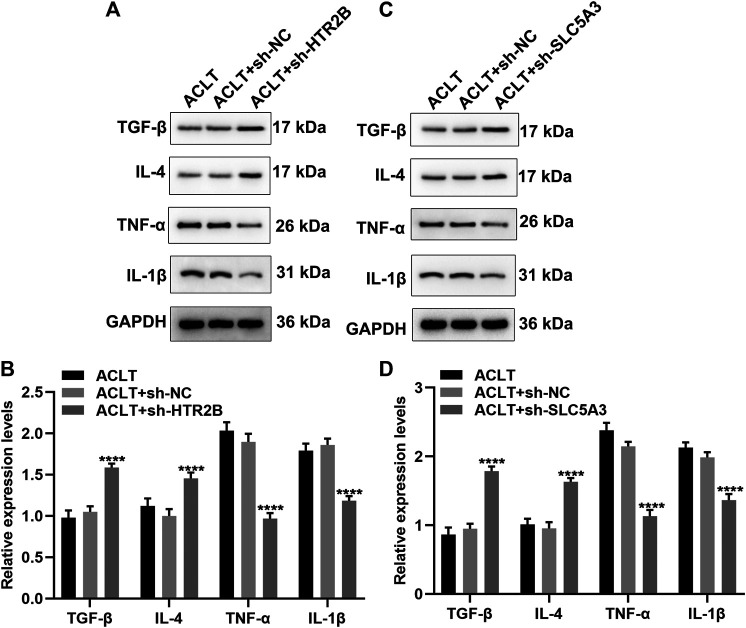
The roles of silencing HTR2B or SLC5A3 on inflammatory response of OA synovial cells. **(A–D)** Western blot for examining the expression of TNF-α, IL-1β, TGF-β and IL-4 in OA synovial cells transfected with **(A,B)** sh-HTR2B or **(C,D)** sh-SLC5A3. ****p < 0.0001.

Synovial inflammation is involved in OA progression. Here, we assessed whether HTR2B or SLC5A3 affected inflammatory response of OA synovial cells. After transfection with sh-HTR2B or sh-SLC5A3, we examined the expression of anti-inflammatory factors (TGF-β and IL-4) and proinflammatory factors (TNF-α and IL-1β) in OA synovial cells. As a result, knockdown of HTR2B or SLC5A3 distinctly increased the expression of TGF-β and IL-4 as well as reduced the expression of TNF-α and IL-1β in OA synovial cells (*p* < 0.05; Figures 9A–D). These data were indicative that silencing HTR2B or SLC5A3 restrained inflammatory response of OA synovial cells.

## Discussion

OA is the most common chronic joint disease in orthopedics, characterized by joint degeneration and synovial and cartilage hyperplasia, which is related to many factors such as age (Liu et al., 2019a; Wang et al., 2019b). It often manifests as articular cartilage degeneration and secondary bone hyperplasia (Sharma, 2021). The traditional concept believes that the biomechanical changes and biochemical factors in the joint cause the destruction of articular cartilage, resulting in OA (Zheng et al., 2021). However, the latest research results indicate that the pathological changes of the synovial tissues may lead to the degeneration of articular cartilage, so the inflammation of the synovial tissues may be the initial link in the onset of OA (Mathiessen and Conaghan, 2017; Wang et al., 2019a; Chou et al., 2020). Here, this study identified two specific markers HTR2B and SLC5A3 in OA synovial tissues. After validation, both could be involved in OA progression.

Traditional biological research focuses on explaining the impacts of individual functional elements (such as DNA, mRNA, and protein) on life activities at the molecular level. Although this method is very important for revealing the genetic mechanism of specific traits, it can only be partially explained the cause of a certain life activity. With the rapid development of sequencing technology, traditional biological research cannot fully and effectively explore the biological significance contained in massive data. As a research method of systems biology, the network uses the data of genome, transcriptome, and metabolome to be widely used in the exploration of life sciences. Compared with other regulatory networks, WGCNA can specifically screen genes related to traits and perform modular classification to obtain co-expression modules with high biological significance (Yin et al., 2018). It has been proven to be an efficient data mining method. Previous studies have applied WGCNA to analyze the pathogenesis of OA. For instance, Zhu et al. identified one OA-related co-expression module and 13 hub genes that were in relation to OA progression by applying WGCNA (Zhu et al., 2020). Gu and colleagues employed WGCNA to find hub genes and pathological processes for OA (Gu et al., 2019). Gao et al. determined key gene modules as well as transcription factors for OA through WGCNA (Gao et al., 2019). Furthermore, Guo and colleagues analyzed gene expression profiling and mined hub genes in subchondral bone of OA (Guo et al., 2018). However, there is currently no research to explore key genes in OA synovial tissues. Here, to fill this gap, we constructed co-expression networks in synovial tissues of OA in the GSE55235 and GSE55457 datasets. By intersecting DEGs and genes in the OA-related modules, we finally identified 17 OA-specific DEGs in synovial tissues. Functional enrichment analysis demonstrated that these genes were mainly related to inflammation-related pathways in OA such as TNF, IL-17, B cell receptor, T cell receptor and Toll-like receptor signaling pathways as well as Th1 and Th2 cell differentiation (Conaghan et al., 2019).

Among 17 OA-specific DEGs, HTR2B and SLC5A3 were up-regulated in end-compared to early-stage OA, indicating that both could be involved in OA progression. Following validation, up-regulated HTR2B and SLC5A3 were found in human OA synovial tissues as well as ACLT-induced OA synovial cells. HTR2B relates to neovascularization and immunomodulation in addition to neurotransmission, indicating that it could be involved in synovitis in the pathogenesis of OA (Z. Liu et al., 2019b; Weber et al., 2019). SLC5A3 encodes sodium/myo-inositol cotransporter 1, related to osteogenesis and bone formation. Subchondral bone plays a pivotal role in OA pathogenesis (Li et al., 2013). Herein, we found that silencing HTR2B and SLC5A3 could ameliorate apoptosis and inflammation of OA synovial cells. Collectively, the two OA-specific genes could participate in OA progression and become promising therapeutic targets for OA.

## Conclusion

Taken together, this study screened two OA-specific markers HTR2B and SLC5A3, which could participate in OA progression. Their overexpression was confirmed in human OA synovial tissues as well as ACLT-induced OA synovial cells. Silencing HTR2B or SLC5A3 remitted apoptosis and inflammation of OA synovial cells. Our findings were indicative that HTR2B and SLC5A3 possessed the potential as therapeutic targets against OA, which required in-depth exploration in further studies.

## Data Availability

The datasets presented in this study can be found in online repositories. The names of the repository/repositories and accession number(s) can be found in the article/Supplementary Material.

## Abbreviations

OA, osteoarthritis; WGCNA, weighted gene co-expression network analysis; GEO, gene expression omnibus; ACLT, anterior cruciate ligament transection; DEGs, differentially expressed genes; FC, fold change; FDR, false discovery rate; TOM, topological overlap matrix; ME, module eigengene; GS, gene significance; MM, module membership; PPI, protein-protein interaction; GO, gene ontology; KEGG, kyoto encyclopedia of genes and genomes; BP, biological process; CC, cellular component; MF, molecular function.
